# Retina‐Inspired Self‐Powered Artificial Optoelectronic Synapses with Selective Detection in Organic Asymmetric Heterojunctions

**DOI:** 10.1002/advs.202103494

**Published:** 2022-01-12

**Authors:** Ziqian Hao, Hengyuan Wang, Sai Jiang, Jun Qian, Xin Xu, Yating Li, Mengjiao Pei, Bowen Zhang, Jianhang Guo, Huijuan Zhao, Jiaming Chen, Yunfang Tong, Jianpu Wang, Xinran Wang, Yi Shi, Yun Li

**Affiliations:** ^1^ National Laboratory of Solid‐State Microstructures, School of Electronic Science and Engineering, Collaborative Innovation Center of Advanced Microstructures Nanjing University Nanjing 210093 P. R. China; ^2^ School of Microelectronics and Control Engineering Changzhou University Changzhou 213164 P. R. China; ^3^ Key Laboratory of Flexible Electronics and Institute of Advanced Materials, Jiangsu National Synergistic Innovation Center for Advanced Materials Nanjing Tech University Nanjing 211816 P. R. China

**Keywords:** artificial optoelectronic synapses, organic asymmetric heterojunctions, selective detection, self‐powered, ultrathin molecular semiconducting crystals

## Abstract

The retina, the most crucial unit of the human visual perception system, combines sensing with wavelength selectivity and signal preprocessing. Incorporating energy conversion into these superior neurobiological features to generate core visual signals directly from incoming light under various conditions is essential for artificial optoelectronic synapses to emulate biological processing in the real retina. Herein, self‐powered optoelectronic synapses that can selectively detect and preprocess the ultraviolet (UV) light are presented, which benefit from high‐quality organic asymmetric heterojunctions with ultrathin molecular semiconducting crystalline films, intrinsic heterogeneous interfaces, and typical photovoltaic properties. These devices exhibit diverse synaptic behaviors, such as excitatory postsynaptic current, paired‐pulse facilitation, and high‐pass filtering characteristics, which successfully reproduce the unique connectivity among sensory neurons. These zero‐power optical‐sensing synaptic operations further facilitate a demonstration of image sharpening. Additionally, the charge transfer at the heterojunction interface can be modulated by tuning the gate voltage to achieve multispectral sensing ranging from the UV to near‐infrared region. Therefore, this work sheds new light on more advanced retinomorphic visual systems in the post‐Moore era.

## Graphical abstract

To approach an almost real retina perception, the first self‐powered optoelectronic synapses are designed that allow for selective detection of UV light from wide spectrum and real time preprocess UV signals without external batteries, which benefit from high‐quality organic asymmetric heterojunctions with flexible band structure engineering.

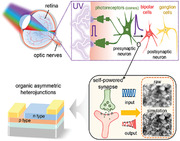

## 1 Introduction

The retina is an essential component of the human visual system, which can perceive nearly 80% of the information from the environment.^[^ ^1^, ^2^ ^]^ In particular, the photoreceptors in the retina can directly detect light of specific wavelengths for the perception of color, and the synapses connecting hierarchical sensory neurons enable real‐time preprocessing.^[^ ^3^, ^4^, ^5^, ^6^, ^7^, ^8^, ^9^, ^10^, ^11^ ^]^ A key superiority of the retina is the selective extraction of core features from massive input visual information, which aims at reducing redundant visual data and accelerating information processing prior to more complicated processing in the brain in an energy‐efficient way.^[^ ^8^ ^]^ Therefore, with the increasing demand for edge computing in the era of big data, various retina‐inspired neuromorphic devices have been proposed to provide a promising pathway toward artificial visual systems with high‐efficiency signal processing.^[^ ^12^, ^13^, ^14^, ^15^, ^16^ ^]^ Recently, optoelectronic synaptic devices with spectral selectivity and multispectral sensing capabilities have attracted considerable attention because they can combine the dual functions of sensing and preprocessing in a single device.^[^ ^1^, ^17^, ^18^, ^19^, ^20^ ^]^ Note that the sustainable retina‐imitating intelligent operation of these devices to cope with ubiquitous sensing still requires an external electrical supply for energy harvesting, energy conversion, and information transmission to obtain useful visual signals.^[^ ^12^, ^21^ ^]^ Consequently, there is a high demand to solve this substantial issue by exploiting rational material matching, device design, and physics to realize a more retina‐like artificial visual system.

Organic thin film devices with unique features of long‐term biocompatibility, molecular diversity, and multiple modes of carrier modulation have shown great potential for bionic retina perception devices.^[^ ^22^, ^23^, ^24^, ^25^, ^26^ ^]^ Herein, we designed self‐powered optoelectronic synapses using organic asymmetric heterojunctions with ultrathin functional molecular semiconducting crystalline films to emulate human retina perception. By virtue of the intrinsic heterogeneous interfaces and typical photovoltaic properties, our devices can selectively detect and preprocess the ultraviolet (UV) light with no battery. Furthermore, multiple self‐powered UV‐light‐modulated short‐term synaptic plasticity are presented, such as excitatory postsynaptic current (EPSC), paired‐pulse facilitation (PPF), and high‐pass filtering characteristics. These zero‐power synaptic operations further facilitate a demonstration of image sharpening functions for image preprocessing. In addition, due to the gate‐tunable charge transfer in the heterojunction, our devices exhibited multispectral sensing capability even beyond that of the photoreceptors in the retina. Therefore, our work opens up a promising path toward a more efficient artificial visual system.

## 2 Results and Discussion

### 2.1 Retina‐Inspired Device Design and Characterizations

**Figure 1:**
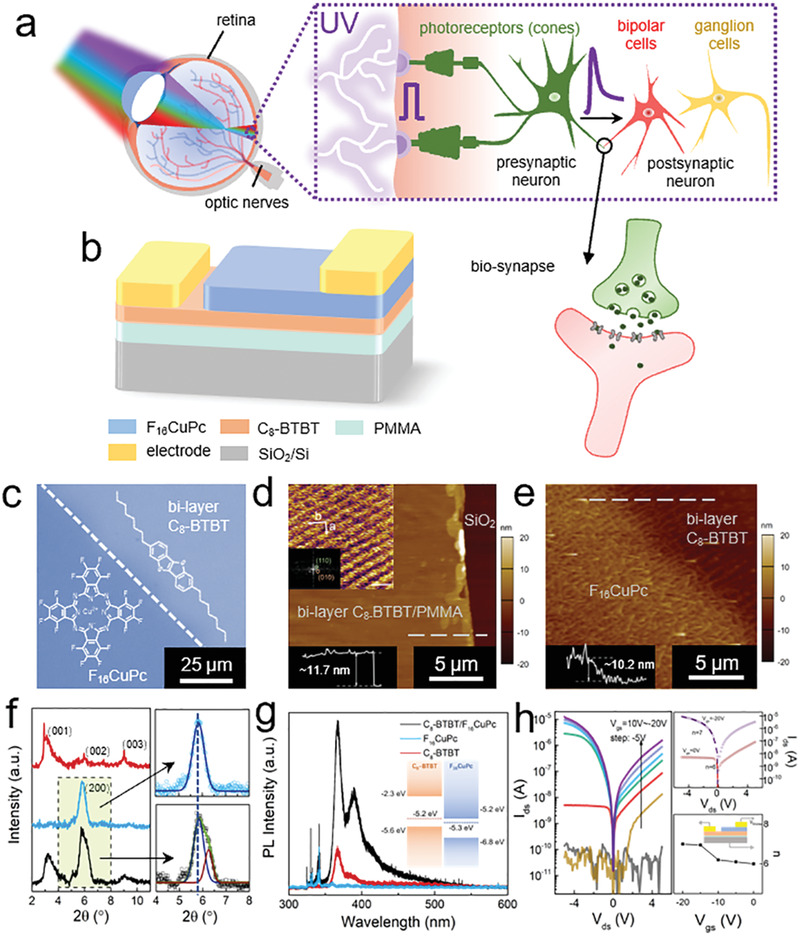
Characterizations of organic asymmetric heterojunctions. a) Schematic of the retina, in which cone cells can detect light of specific wavelengths and synapses readout, process, and memorize the signal from neurons with synaptic plasticity. b) Schematic diagram of the F_16_CuPc/C_8_‐BTBT heterojunction. c) Optical microscopic images of F_16_CuPc/C_8_‐BTBT heterojunctions. The boundaries of the F_16_CuPc and C_8_‐BTBT films are indicated by white dotted lines. The insets show the molecular structures of C_8_‐BTBT and F_16_CuPc. Scale bar, 25 µm. d,e) AFM images of bilayer C_8_‐BTBT films on PMMA films and F_16_CuPc/C_8_‐BTBT heterojunctions, respectively. The height profiles correspond to the gray dotted lines in the AFM images. Scale bar: 5 µm. The inset in (d) is high‐resolution AFM images of C_8_‐BTBT on PMMA films, scale bar: 1 nm. f) XRD patterns of C_8_‐BTBT films (red lines in the left panel), F_16_CuPc films on SiO_2_/Si substrate (blue lines in the left panel), and F_16_CuPc films on C_8_‐BTBT films (black lines in the left panel). The right panels are the fitted (Gaussian) curves of the diffraction peak of F_16_CuPc on SiO_2_/Si substrate (upper) and C_8_‐BTBT films (lower), respectively. g) PL spectra of F_16_CuPc/C_8_‐BTBT heterojunctions and pristine C_8_‐BTBT and F_16_CuPc films under 325 nm excitation light. The inset shows the band alignment of C_8_‐BTBT and F_16_CuPc. h) *I*–*V* curves of the F_16_CuPc/C_8_‐BTBT heterojunction under various gate voltages. The ideality factors *n* extracted from the left panel under different gate voltages. The insets show the measurement setup of the devices.

In the vertebrate retina, abundant sensory neurons connected by synapses are distributed within different layers, mainly including photoreceptors, bipolar cell layers, and ganglion cell layers (**Figure** **1**a).^[^ ^14^ ^]^ Photoreceptors such as rods and cones convert incoming light into electrical signals, especially for cone cells, which can detect light of specific wavelengths.^[^ ^1^ ^]^ Then, the signals are transferred into the bipolar cells by electronic‐to‐ionic conversion.^[^ ^21^ ^]^ Subsequently, synapses play a role in the initial processing and recognition of visual information with synaptic plasticity, which can accelerate perception in the brain.^[^ ^8^ ^]^ Finally, preprocessed visual information is sent to the brain by ganglion cells and optic nerve fibers for further identification and processing.^[^ ^21^ ^]^

To emulate the structures and functionalities of the retina, devices based on semivertical asymmetric organic heterojunctions were fabricated (Figure 1b and Figure S1a–c (Supporting Information), and see the Experimental Section). A double layer structure of the p‐type organic semiconductor dioctylbenzothienobenzothiophene (C_8_‐BTBT) and the dielectric poly(methyl methacrylate) (PMMA) was formed via vertical phase separation, which was demonstrated by the water contact angle measurement (Figure S1g‐i and Note S1, Supporting Information); the PMMA layers were used as the interfacial trapping layer. Subsequently, the n‐type organic semiconductor copper hexadecafluorophthalocyanine (F_16_CuPc) was thermally evaporated onto the C_8_‐BTBT films to form the heterojunctions (Figure 1c). The Raman spectra of the C_8_‐BTBT/F_16_CuPc layered structure and isolated C_8_‐BTBT and F_16_CuPc were first obtained, which showed peaks at the wavelength characteristics for C_8_‐BTBT and F_16_CuPc films (Figure S1e, Supporting Information). Thus, this indicates the formation of C_8_‐BTBT/F_16_CuPc heterojunctions.^[^ ^27^ ^]^ We further characterized the morphologic properties of the heterojunctions *via* atomic force microscopy (AFM) (Figure 1d,e and Figure S1d (Supporting Information)). The results showed that the thicknesses of the C_8_‐BTBT, PMMA, and F_16_CuPc films are ≈5.7, 6.0, and 10.2 nm, respectively. The thickness of the C_8_‐BTBT film indicates a bilayer molecular structure;^[^ ^28^, ^29^, ^30^, ^31^, ^32^ ^]^ additionally, this film on the PMMA thin film exhibits atomic smoothness with a root‐mean‐square roughness of 0.12 nm, reflecting an excellent molecular packing structure. Moreover, high‐resolution AFM (HRAFM) characterizations were performed to evaluate the crystalline properties of 2L C_8_‐BTBT on PMMA films (inset of Figure 1d). The HRAFM images reveal that the 2L molecular molecules orient with a herringbone‐type packing, and the lattice constants are *a* = 8.67 ± 0.13 Å, *b* = 6.40 ± 0.15 Å, and *θ* = 88.75° ± 1.2°. We further characterized the X‐ray diffraction (XRD) of C_8_‐BTBT films, F_16_CuPc films on the SiO_2_/Si substrate, and F_16_CuPc films on C_8_‐BTBT films, which exhibited typical diffraction peak C_8_‐BTBT and F_16_CuPc films (Figure 1f). Compared with the XRD pattern of F_16_CuPc on the SiO_2_/Si substrate, the full width at half maxima of the diffraction peak of F_16_CuPc on C_8_‐BTBT films decreased from 0.59 to 0.51. Interestingly, we further performed AFM measurements of F_16_CuPc films, which were thermally evaporated on both SiO_2_/Si and bilayer C_8_‐BTBT films. F_16_CuPc (10 nm) deposited on the SiO_2_/Si substrate formed small and random spherical grains with high density of grain boundaries (Figure S2a,b, Supporting Information). While, the morphology of F_16_CuPc films (10 nm) on bilayer C_8_‐BTBT films exhibited micrometer‐sized strip‐like crystals, which can be observed in the different regions (Figure S2c–e, Supporting Information). It indicates that the underlying bilayer C_8_‐BTBT films serve as a templating layer for the F_16_CuPc layer.^[^ ^33^, ^34^ ^]^ Therefore, our obtained C_8_‐BTBT/F_16_CuPc heterojunctions exhibit a layered structure, high quality, and crystalline properties with ultrathin thickness.

The highest occupied molecular orbital (HOMO) and the lowest unoccupied molecular orbital (LUMO) energies of C_8_‐BTBT are −5.6 and −2.3 eV, and the corresponding HOMO and LUMO energies of F_16_CuPc are −6.8 and −5.2 eV, respectively, which were measured by the ultraviolet photoelectron spectrometer (inset of Figure 1g and Figure S3 and Note S2 (Supporting Information)).^[^ ^35^, ^36^ ^]^ Therefore, the heterojunctions have a type‐II energy band alignment. To further characterize the interfacial charge transfer properties of the heterojunctions, photoluminescence (PL) measurements were performed on the heterojunctions, pristine C_8_‐BTBT, and F_16_CuPc films (Figure 1g). In the C_8_‐BTBT/F_16_CuPc heterojunctions, a PL intensity enhancement was observed. We considered that this phenomenon resulted from the photoexcited holes being withdrawn from C_8_‐BTBT to F_16_CuPc (inset of Figure 1g).^[^ ^37^ ^]^ Additionally, the UV–vis absorption spectrum of the heterojunction combined the absorption characteristics of both materials and showed broad spectral response covering UV, visible, and near‐infrared (NIR) regions with a maximum absorption peak at ≈365 nm (Figure S1f, Supporting Information). Hence, our asymmetric heterojunctions with interface charge transfer properties potentially enable the realization of more advanced optoelectrical applications.

Subsequently, the electrical characteristics of our devices were measured under various gate voltages (Figure 1h). For the measurement setup, the source electrode on C_8_‐BTBT was kept grounded, and a bias voltage (*V*_ds_) was applied to the drain electrode on F_16_CuPc in all the measurements (inset in Figure 1h). Under a negative bias, electrons and holes were injected and accumulated in F_16_CuPc and C_8_‐BTBT, respectively. We fitted the current curves by the Shockley diode equation^[^ ^38^ ^]^

(1) $$I_{\mathrm{ds}}=\left(nV_{T}\right)/R_{s}W\left[I_{0}R_{s}/\exp\left(\left(V_{\mathrm{ds}}+I_{0}R_{s}\right)/\left(nV_{T}\right)\right)\right]-I_{0}$$

where *n* is the ideality factor, *V*_T_ = *k*_B_ *T*/*e* is the thermal voltage, *k*_B_ is the Boltzmann constant, *e* is the elementary charge, *I*_0_ is the reverse bias current, *R*_s_ is the series resistance, and *W* is the Lambert *W* function. In our heterojunctions, an ideality factor of *n* = 6 was obtained when *V*_gs_ = 0 V (for an ideal p–n junction, *n* = 1). Considering the ultrathin organic thin films in our heterojunctions, the charge transport at a small negative bias regime is mainly dominated by the interlayer recombination current (Shockley–Read–Hall and Langevin recombination), because of the trap states acting as recombination centers.^[^ ^38^, ^39^, ^40^ ^]^ The large output current occurs when applying a positive bias voltage, which is quite distinct from that in the common p–n junction. Such behaviors can be attributed to hole tunneling through the ultrathin F_16_CuPc film, which is further confirmed using the Fowler–Nordheim model (Figure S4 and Note S3, Supporting Information).^[^ ^41^, ^42^ ^]^ In addition, we measured the transfer characteristics of our asymmetric‐heterojunction‐based devices (Figure S5, Supporting Information). It shows obvious hysteresis, which indicates the charge trapping effect of the PMMA layers.

### 2.2 Photovoltaic Properties of Self‐Powered Optoelectronic Synapses

**Figure 2:**
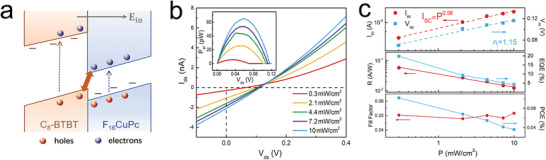
Photovoltaic response properties of organic asymmetric heterojunctions. a) Energy band diagram of the F_16_CuPc/C_8_‐BTBT heterojunction under 365 nm light illumination without a power supply. b) *I*–*V* curves under various light intensities. The inset shows the electric power generated by the heterojunction device versus *V*_ds_ under various incident light powers. c) Light‐power‐dependent short‐circuit current *I*_sc_ and open‐circuit voltage *V*_oc_ (upper panel). The fitting curves use the equations *I*_sc_ ∝ *P^*α*^* and (d*V*_oc_)/dlog*P* = *n*_i_ *kT*/*q*, respectively. Light‐power‐dependent responsivity and EQE versus incident light power (middle panel). The fitting curve uses the equation *R* ∝ *P* ^−^ *^*α*^*. Fill factor and PCE versus incident light power for the F_16_CuPc/C_8_‐BTBT heterojunction (lower panel).

The prerequisite for self‐powered optoelectronic synaptic devices is the photonic‐to‐electronic conversion capability without any electrical power supply.^[^ ^21^, ^22^ ^]^ Our asymmetric heterojunctions with type‐II band alignment are promising to fabricate self‐powered optoelectronic devices. We first obtained *I*_ds_–*V*_ds_ curves of our asymmetric heterojunction devices under 365 nm illumination of various intensities (from 0.3 to 10 mW cm^−2^) (**Figure** **2**b). The curves are all downshifted, exhibiting a distinct photovoltaic response. This can be further explained by the energy band diagram of our heterojunctions (Figure 2a). When the devices are under light illumination, photoinduced electron–hole pairs were generated and separated in the heterojunction under the large built‐in electric field. Note that, in our asymmetric heterojunction architecture, holes were rapidly collected on the F_16_CuPc side due to the short diffusion distance of the vertical junction, and electrons crossed the heterointerface and arrived at the C_8_‐BTBT side.^[^ ^43^ ^]^ Therefore, a positive open‐circuit voltage (*V*_oc_) of 116 mV and a negative short‐circuit current (*I*_sc_) of ≈2.24 nA were observed at a light power of 10 mW cm^−2^ (Figure 2b). Besides, we found a polarity‐reversible photocurrent response under various gate voltages, which can be associated with gate‐tunable band alignment under the ultrathin organic functional semiconducting films (Figure S6 and Note S4, Supporting Information). Therefore, our devices with an asymmetric heterostructure exhibit a typical photovoltaic response.

To quantify the photovoltaic response performance, the power‐dependent photoresponse of the heterojunctions was further measured. According to the upper panel of Figure 2c, *I*_sc_ exhibited a sublinear relationship with the light power, described by the power law *I*_sc_ ∝ *P^*α*^*, where the power exponent *α* is equal to 0.56. In general, the exponent 0 < *α* < 1 indicates that the photogating effect contributes to the photocurrent in our devices.^[^ ^44^, ^45^ ^]^ Besides, *V*_oc_ also increased with the light power, fitted by the equation (d*V*_oc_)/dlog*P* = *n*_i_ *kT*/*q*, in which *n*_i_ is the ideality factor. An ideality factor of *n*_i_ = 1.15 was obtained in our device, illustrating that the Langevin process results in recombination.^[^ ^38^, ^41^ ^]^ The middle panel of Figure 2c shows the responsivity (*R*) of our device at different light powers, defined as the ratio of the photocurrent (*I*_ph_) to the incident light power (*P*). The device demonstrated a maximum *R* of 58 mA W^−1^ at a light power of 0.3 mW cm^−2^. Moreover, another figure of merit is the external quantum efficiency (EQE), expressed as EQE = *Rhc*/*eλ*, where *h* is the Planck's constant, *c* is the speed of light, *e* is the elementary charge, and *λ* is the wavelength of the incident light. The highest EQE of ≈19% is obtained at a light power of 0.3 mW cm^−2^. The output electric power *P*_el_ = *I*_ds_ *V*_ds_ as a function of *V*_ds_ was plotted in the inset of Figure 2b. The fill factor FF = *P*_el,max_/(*I*_sc_ *V*_oc_) (*P*_el,max_ is the maximum power point of *P*_el_) and the power conversion efficiency PCE = *P*_el,max_/*P* were displayed in the lower panel of Figure 2c. Both FF and PCE increased with increasing incident power. Besides, we also compared the photovoltaic performance of heterojunctions using F_16_CuPc with different thicknesses. Confined to the exciton diffusion length (≈10 nm), devices with F_16_CuPc with 10 nm demonstrate the highest short current level, which are the optimal choice in our heterojunction devices (Figure S7, Supporting Information).

### 2.3 Self‐Powered UV‐Light‐Modulated Synaptic Behaviors

Based on the photovoltaic properties of our asymmetric heterojunctions that can act as an internal power source,^[^ ^21^, ^22^ ^]^ we achieved self‐powered synaptic behaviors to emulate the optic‐neural synapses in the retina. A typical EPSC response under UV pulse (365 nm, 3 mW cm^−2^, 50 ms) can be observed in a single device, which is similar to the short‐term plasticity in biological synapses (Figure S8 and Note S5, Supporting Information). Compared with other low‐power optoelectronic synaptic devices still driven by a small electrical bias, our devices can work without power consumption for power‐efficient visual perception.^[^ ^20^, ^23^, ^25^ ^]^ The asymmetric heterojunction architecture is a key to realize the self‐powered EPSC response, the short distance between C_8_‐BTBT and F_16_CuPc benefits the collection of the photogenerated holes after being separated at interface, and the lateral channel of C_8_‐BTBT can contribute to the synaptic behaviors after the light pulse ends due to the photogenerated electron trapping at the C_8_‐BTBT/PMMA interface (Figure S9 and Note S6, Supporting Information).^[^ ^46^, ^47^, ^48^ ^]^ Note that in a self‐powered mode, our synaptic devices can only detect and preprocess the UV light, exhibiting wavelength selectivity (Figure S10 and Note S7, Supporting Information). Besides, our devices exhibited good repeatability and synaptic performance in a self‐powered mode (Figure S11, Supporting Information). In addition, considering that high stability is still a challenge for devices using ultrathin organic molecular crystals, encapsulation is a promising approach to guarantee much reliable and stable device performance (Figure S12, Supporting Information).

**Figure 3:**
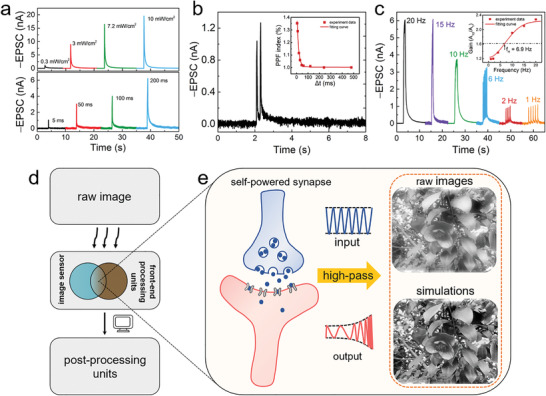
Self‐powered light‐tunable synaptic characteristics and image preprocessing simulation. a) EPSC triggered by various light intensities and durations. b) EPSC triggered by one pair of light pulses with a duration of 20 ms and a Δ*t* of 20 ms. The PPF index, defined as *A*_2_/*A*_1_ × 100%, is plotted and fitted as a function of Δ*t* in the inset. c) EPSC triggered by ten continuous light pulses with a duration of 50 ms under various pulse rates. The gain, defined as *A*_10_/*A*_1_ × 100%, is plotted and fitted as a function of light pulse frequency in the inset. d) Schematic diagram of an artificial visual system in which the front‐end processing units are integrated in the image sensors. e) Schematic diagram of high‐pass filtering of the self‐powered synapse. The insets are the images before (upper) and after (lower) image sharpening.

Furthermore, varying degrees of short‐term plasticity in self‐powered optoelectronic synapses were demonstrated. Our devices were examined under light pulses with different intensities (from 0.3 to 17 mW cm^−2^) (the upper panel of **Figure** **3**a), exhibiting light‐intensity‐dependent characteristics similar to those of conventional Si‐based image sensors. Note that complicated processing functions often require time sensitivity, and we further studied the illumination‐time‐dependent self‐powered EPSC response (the lower panel of Figure 3a).^[^ ^49^ ^]^ When applying relatively short UV pulses (<100 ms), the response time of our synaptic devices (the time intervals for the response to rise from 10% to 90% of the current under light illumination) is quite close to the duration time of the UV pulse. And the current variation can exhibit typical and obvious synaptic behaviors when the UV pulse lasts 50 ms. Besides, further enlarging the duration time of the UV pulses allows the current increase to a saturated value yet within a short response time of ≈160 ms (the green line in Figure S13a in the Supporting Information). By utilizing a relatively weak light pulse, a small current peak was induced by a small amount of photogenerated carriers and quickly decayed to the initial state. By contrast, a long illumination time gave rise to an enhanced EPSC and a long relaxation time, which can be understood as a result of the large amount of photogenerated carriers leading to more trapped photoinduced electrons. We also studied the response time of our self‐powered synapses versus duration time of UV pulses under different channel lengths (Figure S13 and Note S8, Supporting Information). The limited response time is ≈5 ms, which is comparable to that of biological retina cells.^[^ ^8^, ^50^, ^51^ ^]^ In addition, because of the blurring effect during the F_16_CuPc deposition, the ambipolar transport behavior appeared when the device footprint (channel length) downscaled to 10 µm (i.e., without the self‐powered synaptic response).^[^ ^52^ ^]^ Also, the time‐dependent plasticity enabled learning and forgetting functions, further confirming significant sensitivity to optical signals (Figure S14 and Note S9, Supporting Information).

In biology, PPF is sensitive to the time‐series information, which can be used to recognize and decode temporal information.^[^ ^19^ ^]^ The PPF behaviors of self‐powered synapses can be mimicked by applying two consecutive UV pulses with a time interval (Δ*t*) of 20 ms (365 nm, 1.2 mW cm^−2^, 20 ms), and the response to the two consecutive pulses is shown in Figure 3b. The amplitude of the second EPSC (*A*_2_) was obviously larger than that of the first EPSC (*A*_1_), indicating that some of the trapped photogenerated electrons triggered by the first pulse cannot be fully released before the second pulse arrives, which further demonstrates the time‐dependent characteristics. Subsequently, we plotted the PPF ratio (*A*_2_/*A*_1_) versus Δ*t* in the inset of Figure 3b. The time‐interval‐dependent PPF ratio can be fitted by a biexponential decay function^[^ ^53^, ^54^ ^]^

(2) $$\mathrm{PPF}\;\mathrm{ratio}=1+C_{1}\exp\left(-\Delta t/\tau_{1}\right)+C_{2}\exp\left(-\Delta t/\tau_{2}\right)$$

where Δ*t* is the time interval between the pair of light stimuli, *τ*_1_ and *τ*_2_ are the characteristic relaxation times of the two phases, *C*_1_ and *C*_2_ are the initial facilitation values of the rapid and slow phases, respectively. *C*_1_ = 0.15, *C*_2_ = 0.203, *τ*_1_ = 10 ms, and *τ*_2_ = 1200 ms are extracted by fitting Equation (2). The obtained short and long relaxation times are similar to those in biological synapses.^[^ ^55^ ^]^ As a result, PPF behaviors further demonstrate the time‐dependent plasticity of our self‐powered optoelectronic synaptic devices.

Image preprocessing can selectively extract useful data from extensive raw data by enhancing the feature for further processing, which is a main characteristic of the human retina.^[^ ^49^, ^56^ ^]^ Note that synaptic devices with short‐term plasticity can collect and refine massive perception information in signal transmission, which play a critical role on image preprocessing in the intelligent sensory system.^[^ ^9^, ^10^, ^11^ ^]^ By virtue of light‐intensity‐dependent and illumination‐time‐dependent plasticity, our self‐powered optoelectronic synapses have the potential to perform dynamic high‐pass filtering operations for image preprocessing. Biologically, synapses with a low initial probability of vesicle release in neuroscience can act as high‐pass filters for information processing depending on the frequency of the stimulation.^[^ ^57^ ^]^ To study the filtering properties of our self‐powered synapses, a series of UV pulse trains (365 nm, 1.2 mW cm^−2^, 50 ms) with different frequencies were applied to the device to evaluate the EPSC of the device, and each stimulus train contained 10 light pulses (Figure 3c). The amplitude gain is defined as *A*_10_/*A*_1_, where *A*_10_ and *A*_1_ are the amplitudes of the tenth EPSC and the first EPSC, respectively. The gain, plotted as a function of pulse frequency, increased from 1 to 2.32 with increasing frequency from 1 to 20 Hz (inset in Figure 3c). Furthermore, the pulse‐frequency‐dependent gain was fitted with a high‐pass filter function described by $H=1-\exp(-f^{2}/\left(2f_{H}^{2}\right))$,^[^ ^58^ ^]^ where *f* is the frequency of the input light pulse and *f*_H_ is the cutoff frequency; a *f*_H_ of 6.9 Hz was obtained. For a high‐pass filter, the signals can only pass through the filter if *f* is higher than *f*_H_, and this property can be used for image preprocessing to realize edge enhancement, i.e., image sharpening. In order to show the dynamic filtering properties more vividly, a simulation of the high‐pass filter was performed on a raw image of flowers with a weak contrast (Figure 3e). When the *f*_H_ was utilized for high‐pass filters, the flowers in the preprocessed image were sharpened, which exhibited obvious edge enhancement (detailed process in the Experimental Section). This simulation result reveals that our self‐powered asymmetric‐heterojunction‐based synapses integrating image sensing and front‐end processing in a single device have the potential to provide a simple geometry with in‐sensor computing capabilities for image preprocessing in visual biological applications (Figure 3d and Figure S15 and Note S10 (Supporting Information)).

### 2.4 Gate Tunability and Multispectral Sensing beyond the Retina Perception

**Figure 4:**
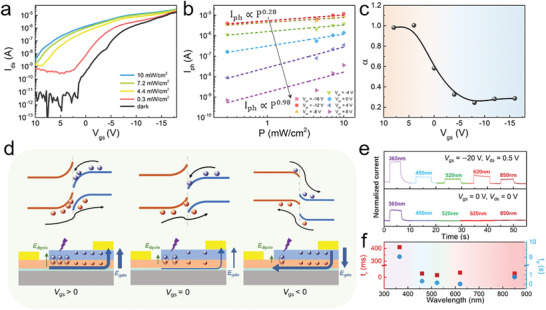
Gate tunability and multispectral sensing. a) Transfer curves of the F_16_CuPc/C_8_‐BTBT heterojunction in the dark and under various light intensities. b) Photocurrent plotted and fitted as a function of light power under different gate voltages. c) *α* extracted in fitting of the light intensity dependence under various gate voltages. d) Schematic illustration of the band diagram and heterojunction operation under *V*_gs_ > 0 V (left), *V*_gs_ = 0 V (middle), and *V*_gs_ < 0 V (right). e) Transient photocurrent under various light wavelengths at *V*_gs_ = −20 V (upper panel) and *V*_gs_ = 0 V (lower panel). f) Response time versus incident light wavelength.

Multispectral sensing and spectral selectivity capabilities achieved in a single optical‐sensing device enable accurate detection and better object identification under complex conditions (Note S11, Supporting Information).^[^ ^59^ ^]^ As stated above, our asymmetric heterojunctions can selectively detect and process UV light in a self‐powered mode due to the intrinsic energy band structures (lower panel of **Figure** **4**e). Furthermore, we demonstrated the tunable charge transfer of our asymmetric heterojunctions under external gate voltages for multispectral sensing, which could provide an opportunity to expand the functionalities for more realistic emulation of a retina.

The transfer curves under dark conditions and various light intensities at 365 nm under *V*_ds_ = −15 V are shown in Figure 4a. We extracted and fitted the photocurrent (*I*_ph_ = *I*_light_ − *I*_dark_) as a function of incident light power under different gate voltages (*I*_ph_ ∝ *P^*α*^*) (Figure 4b). A transition in which *α* changes from ≈1 (photoconduction) to <1 (photogating) under varied gate modulations from 8 to −16 V was found (Figure 4c), which is ascribed to the gate‐modulated interfacial charge transfer dipole between C_8_‐BTBT and F_16_CuPc (Figure 4d).^[^ ^60^, ^61^ ^]^ The initial interfacial dipole stems from the hole transfer from F_16_CuPc to C_8_‐BTBT at *V*_gs_ = 0 V. When a negative gate bias (*V*_gs_ < 0 V) is applied, the direction of the electric field is different from that of the interfacial dipole, which can be beneficial to charge transfer at the type‐II interface (the right inset of Figure 4d).^[^ ^61^ ^]^ A table that comprehensively summarizes the changes of the interfacial dipole and band alignment is shown in Table S1 (Supporting Information). By virtue of the absorption characteristics of the heterojunction, this gate tunability allows our devices with the ability of the multispectral sensing. The photoswitching characteristics of our device at *V*_gs_ = −20 V show increasement of photocurrent from UV to NIR regions (upper panel of Figure 4e). Furthermore, the rise (*t*_r_)/decay (*t*_d_) times, defined as the time intervals for the response to rise/decay from 10%/90% to 90%/10% of the drain current under light illumination, were extracted from the photoswitching curves (Figure 4f). The shortest *t*_r_ (*t*_d_) is only ≈9 ms (20 ms) in the green (red) light region, and we considered that the longer response time in the UV light region was due to the photogenerated carriers trapped by deep‐level traps under the synergism of high‐energy UV light and drain voltage (Figure S16 and Note S12, Supporting Information). In addition, our devices can facilitate wider dynamic and more intelligent neural functions such as multimode logic computation beyond those of conventional simple synapse‐level optoelectronic devices (Figure S17 and Note S13, Supporting Information). Therefore, our asymmetric heterojunctions can realize UV‐light‐modulated self‐powered synaptic behaviors and multispectral sensing ranging from UV to NIR regions under gate voltages, which can exhibit more comprehensive perception abilities for more accurate information detection and processing to emulate the superior neurobiological features of the human retina.

## 3 Conclusion

We demonstrated self‐powered optoelectronic synapses utilizing organic asymmetric heterojunctions with ultrathin functional layers, which successfully reproduce the unique connectivity among sensory neurons and even transcend the sensing functions of the retina. Our devices exhibit an obvious photovoltaic effect, which can act as a power supply. More importantly, combining this capability and interface charge trapping by coupling the heterogeneous interfaces gives rise to selective detection of UV light and self‐powered synaptic behaviors with no battery. Furthermore, this zero‐power optical‐sensing synaptic plasticity allows us to perform first‐stage image preprocessing, i.e., an image sharpening function. Additionally, the gate‐tunable charge transfer at the heterojunction interface can contribute to multispectral sensing ranging from the UV to NIR region. Therefore, such satisfactory results can afford a bright future for energy‐efficient artificial visual systems that will significantly promote the development of edge computing.

## 4 Experimental Section

### Fabrication of Semivertical Crystalline F_16_CuPc/C_8_‐BTBT Heterojunctions

C_8_‐BTBT (≥99%) and F_16_CuPc (≥80%) were purchased from Sigma‐Aldrich and used without further purification. C_8_‐BTBT (0.5 wt%) and PMMA (0.1 wt%) were dissolved in a mixture of anisole and *p*‐anisaldehyde (0.5 wt%). Subsequently, 3.5 µL of the solution was dropped onto a precleaned 50 nm SiO_2_/Si substrate, which was cleaned in acetone, isopropanol, and deionized water for 15 min each by sonication. Subsequently, the antisolvent‐assisted crystallization technique was adopted, and a double layer structure of C_8_‐BTBT and PMMA was formed via vertical phase separation.^[^ ^62^, ^63^ ^]^ F_16_CuPc was then thermally evaporated as the n‐type semiconductor onto the C_8_‐BTBT films with a shadow mask. The evaporation pressure was 5 × 10^−4^ Torr. The deposition rates of F_16_CuPc were kept at 0.1 Å s^−1^, which was monitored by a quartz crystal oscillator.

### Characterizations of Semivertical Crystalline F_16_CuPc/C_8_‐BTBT Heterojunctions

For regular AFM and HRAFM, the characterizations were performed with an Asylum Research Cypher scanning probe microscope under ambient conditions. Raman spectroscopy was performed using a COHERENT system. The Raman system was equipped with a confocal microscope and a charge‐coupled device Si detector. A 485 nm laser was used as the excitation light source for Raman spectroscopy. PL spectroscopy was performed using a LabRAM HR800. A 325 nm laser was used as the excitation light source, which was focused onto the sample through an objective (100×) to an excitation spot. A Rigaku Smartlab X‐ray diffractometer was used to perform the XRD.

### Device Fabrication

After the film of the heterojunction was deposited, two Au electrodes (thickness of 100 nm) with an area of 80 × 120 µm^2^ were transferred onto its surface by “stamping” Au stripes with channel width and length of ≈90 and 20 µm, respectively. Notably, one of the two Au electrodes was attached to the 2L C_8_‐BTBT film, and the other electrode was attached to the F_16_CuPc film, which was next to the 2L C_8_‐BTBT film.

### Electrical and Optoelectrical Measurements of F_16_CuPc/C_8_‐BTBT Heterojunctions

Electrical measurements were carried out with a semiconductor parameter analyzer (Agilent B1500) in a closed‐cycle cryogenic probe station with a vacuum condition of 10^−3^ Torr. A 365 nm UV‐light‐emitting diode (LED) (450, 520, 620, and 850 nm LEDs for multispectral sensing) driven by a signal generator was used as a light source to illuminate F_16_CuPc/C_8_‐BTBT heterojunction photodiodes from the top. The 365 nm UV LED light was calibrated by a UV‐A meter (LS125, UVALED‐X3 probe).

### Simulation of Image Sharpening

For the simulation of the high‐pass filter on the self‐powered optoelectronic synapses, an image of the flowers was treated as an example with the assistance of the MATLAB. First, a matrix of an input flower image with a size of *m* × *n* (the time‐domain information) was given, which was transformed from Red‐green‐blue mode to grayscale mode. Then, the time‐domain signal was transformed into the frequency‐domain signal by using a 2D Fourier transform. Subsequently, the Fourier shift was used to move the low‐frequency signal with *f* = 0 to the center of the matrix (*u* = *m*/2, *v* = *n*/2). After the first two steps, a matrix of the same size as the original image was obtained that contained the amplitude and frequency information of the original image, denoted as *F*(*u*, *v*). A high‐pass filter $H\left(u,v\right)=1-\exp\left(-f^{2}/\left(2f_{H}^{2}\right)\right)$ was used based on the filtering characteristics of the synaptic devices. Here, *f* ^2^  =  (*u*  −  *m*)^2^  +  (*v*  −  *n*)^2^. The cutoff frequency of our device was 6.9. The filtering was carried out by *G*(*u*, *v*) = *H*(*u*, *v*)*F*(*u*, *v*). The final image could be obtained by applying the inverse Fourier transform.

### Statistical Analysis

All shown data are representative for the samples. Among these data, Figure 4e used normalized processing. And MATLAB was utilized for simulation of image sharpening.

## Conflict of Interest

The authors declare no conflict of interest.

## Supporting information

Supporting Information

## Data Availability

Research data are not shared.
