# Supplementary material for: Retina‐Inspired Self‐Powered Artificial Optoelectronic Synapses with Selective Detection in Organic Asymmetric Heterojunctions
Source: Adv Sci (Weinh). 2022 Jan 12;9(7):2103494. doi: 10.1002/advs.202103494 (PMC8895149; doi:10.1002/advs.202103494)
Supplement: Supplementary file 1 — Supporting Information [file ADVS-9-2103494-s001.pdf]

ADVANCED SCIENCE
Open Access

Supporting Information

for *Adv. Sci.,* DOI: 10.1002/advs.202103494

Retina-inspired self-powered artificial optoelectronic synapses with selective detection in organic asymmetric heterojunctions

Ziqian Hao, Hengyuan Wang, Sai Jiang, Jun Qian, Xin Xu, Yating Li, Mengjiao Pei, Bowen Zhang, Jianhang Guo, Huijuan Zhao, Jiaming Chen, Yunfang Tong, Jianpu Wang, Xinran Wang, Yi Shi, and Yun Li**

Supporting Information

Retina-inspired self-powered artificial optoelectronic synapses with selective detection in organic asymmetric heterojunctions

Ziqian Hao, Hengyuan Wang, Sai Jiang, Jun Qian, Xin Xu, Yating Li, Mengjiao Pei, Bowen Zhang, Jianhang Guo, Huijuan Zhao, Jiaming Chen, Yunfang Tong, Jianpu Wang, Xinran Wang, Yi Shi, and Yun Li**

Z. Hao, H. Wang, Dr. J. Qian, X. Xu, Y. Li, M. Pei, B. Zhang, J. Guo, Dr. H. Zhao, J. Chen, Prof. X. Wang, Prof. Y. Shi, Prof. Y. Li
National Laboratory of Solid-State Microstructures, School of Electronic Science and Engineering, Collaborative Innovation Center of Advanced Microstructures
Nanjing University
Nanjing 210093, P. R. China.
E-mail: yshi@nju.edu.cn; yli@nju.edu.cn

Dr. S. Jiang
School of Microelectronics and Control Engineering
Changzhou University
Changzhou 213164, P. R. China

Y. Tong, Prof. J. Wang
Key Laboratory of Flexible Electronics and Institute of Advanced Materials, Jiangsu National Synergistic Innovation Center for Advanced Materials
Nanjing Tech University
Nanjing 211816, P. R. China

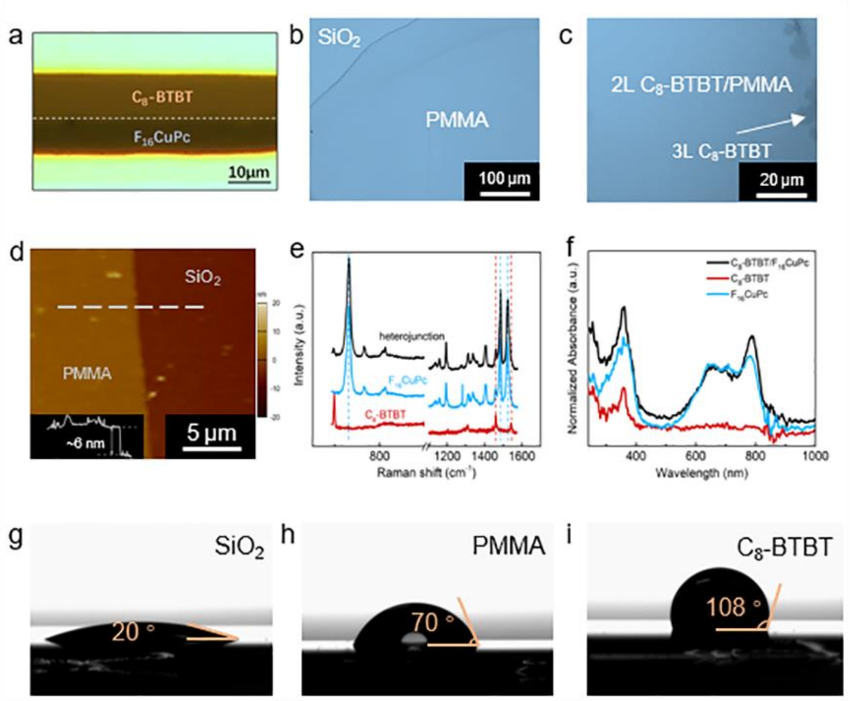

Figure S1. a) The optical image of the device with electrodes. b, c) The optical images of PMMA films after removing the C_8-BTBT with cyclohexane and bilayer C_8-BTBT films on PMMA films, respectively. d) AFM images of PMMA films. The height profiles correspond to the grey dotted lines in the AFM images. e) Raman shift of F_{16}CuPc/C_8-BTBT heterojunctions, the pristine C_8-BTBT and F_{16}CuPc films under excitation light of 485 nm. f) The absorption spectra of F_{16}CuPc/C_8-BTBT heterojunctions, the pristine C_8-BTBT and F_{16}CuPc films. g-i) Water contact angles of bare SiO_2, PMMA films, and C_8-BTBT films, respectively.

Note S1. The measurement of water contact angles.

We confirmed the film structure by measuring water contact angles. According to Figures S1g-i, the contact angle of the film increases in the sequence of SiO_2 (~20°), PMMA (~70°), and C_8-BTBT (~108°). Therefore, this result can further demonstrate the phase-separated structure of the blend film (the PMMA layer is the bottom layer and the C_8-BTBT layer is the top layer).

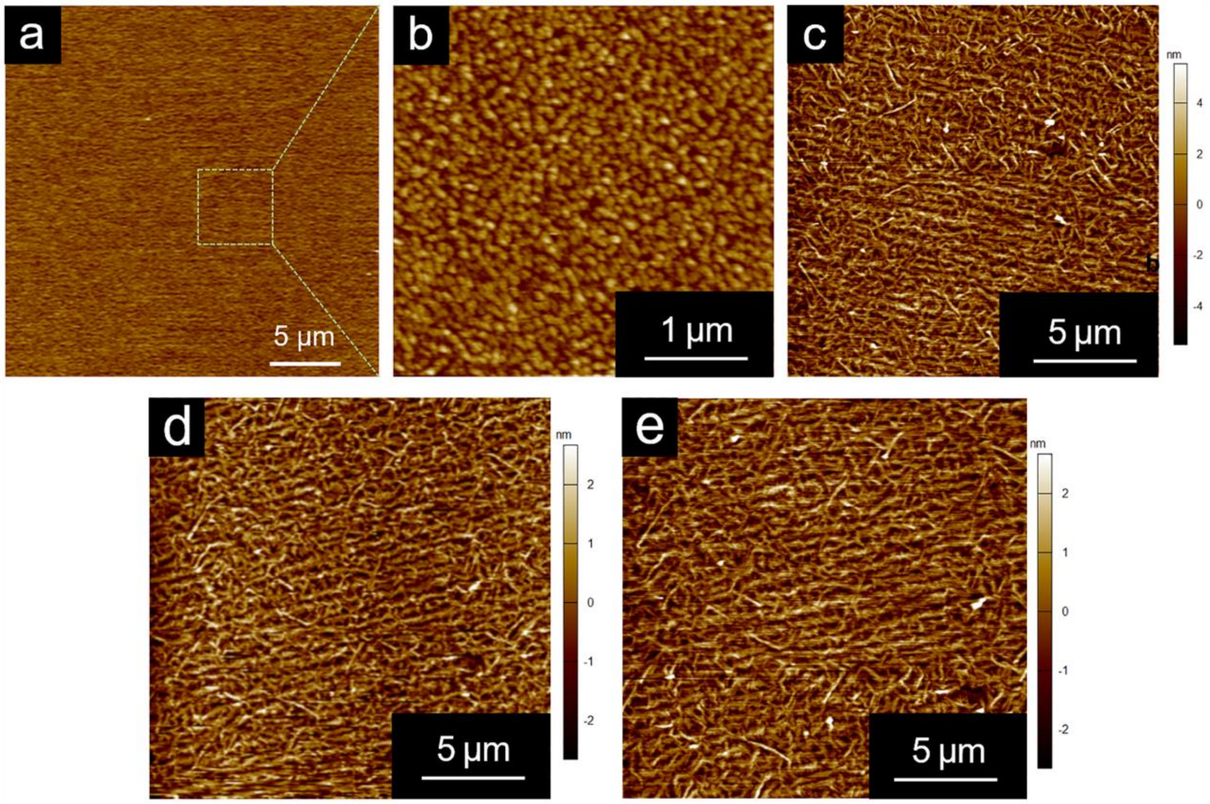

Figure S2. a, b) The AFM images of 10 nm $F_{16}CuPc$ on the Si/SiO_2 substrate. c-e) The AFM images of 10 nm $F_{16}CuPc$ on the C_8-BTBT films appearing in the different regions.

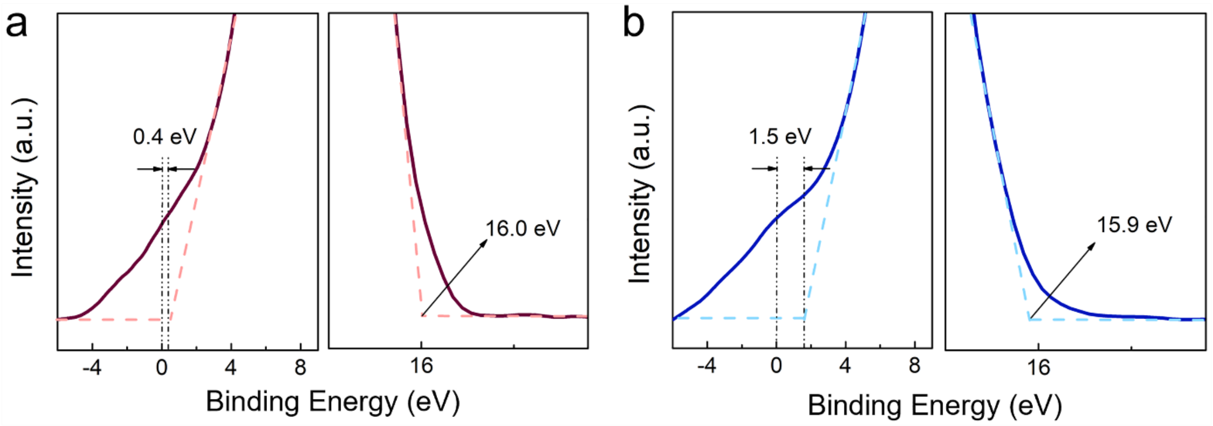

Figure S3. UPS spectra of bare C_8-BTBT (a) and F_{16}CuPc (b) displaying the valence band spectra and second electron cutoffs, respectively.

Note S2. The measurement of the ultraviolet photoelectron spectrometer.

The ultraviolet photoelectron spectrometer (UPS) was measured to re-determine energy band alignment of C_8-BTBT/F_{16}CuPc heterostructure (Figure S3). The measured work functions (W) of C_8-BTBT and F_{16}CuPc were 5.2 and 5.3 eV, respectively ($W = h\nu - E_{cut}$, where $h\nu$ = 21.2 eV is the photon energy of He I light source).[1] Furthermore, the highest occupied molecular (HOMO) of C_8-BTBT and F_{16}CuPc were calculated to be 5.6 and 6.8 eV, respectively ($E_{HOMO} = h\nu - (E_{cut} - E_{VB})$).[2] In addition, according to the PL measurement and reports of the bandgaps of C_8-BTBT (~3.3 eV) and F_{16}CuPc (~1.6 eV), a type-II band alignment can be confirmed (inset of Figure 1c).[3] Note that since C_8-BTBT exhibits a lower work function, electrons can be transferred to F_{16}CuPc when they are brought in contact.[1] Under 325 nm light illumination, the photoexcited holes will transfer from C_8-BTBT (p-type) to F_{16}CuPc (n-type) under the built electric field. Therefore, we considered that obvious PL enhancement in our work originated from photoexcited holes being withdrawn from C_8-BTBT to F_{16}CuPc.

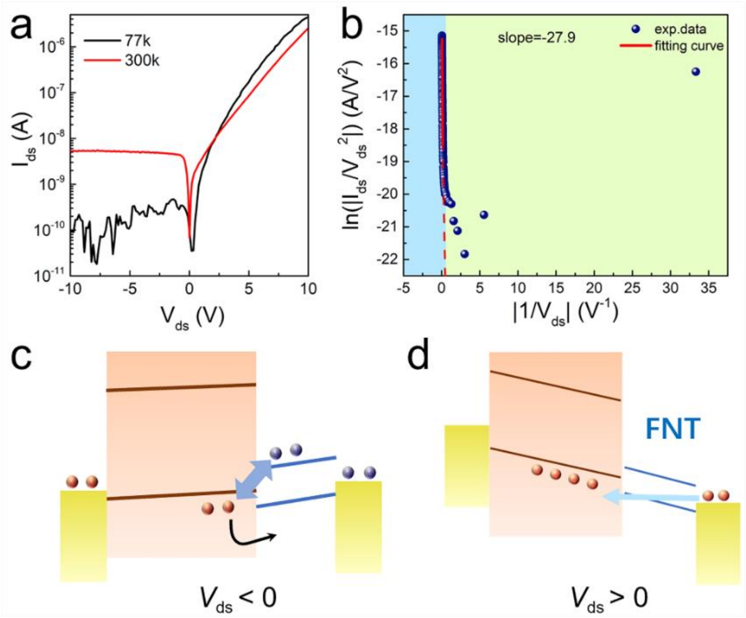

Figure S4. a) The output curves under V_{gs} = 0 V at 77 K (black curve) and 300 K (red curve). b) Fowler–Nordheim plots of the heterojunction under positive bias voltages at 77 K. c-d) The band diagrams of the heterojunction under the negative (c) and positive (d) bias voltages.

Note S3. Electrical properties of our asymmetric heterojunctions.

We measured the current-voltage (I_{ds}-V_{ds}) output characteristics of the asymmetric heterojunctions under V_{gs} = 0 V at 300 K and 77 K (Figure S4a). The devices showed an asymmetrical electrical behaviour at 300 K, which became more striking at 77 K. Under small negative bias, the electrons (holes) were accumulated in F_{16}CuPc (C_8-BTBT). In this case, the heterojunction had a type-II band alignment and the current was dominated by the interlayer recombination of the charge carriers and over-barrier free-hole transport, which reduced significantly as the temperature decreased (temperature-dependent) (Figure S4c).[4] While applying a positive bias, a different dependence was observed in which the current increased under the high bias region.[4] Electrons (holes) were depleted in F_{16}CuPc (C_8-BTBT), and a large electric field drop occurred across the heterojunction, leading to a severe band bending. At the same time, with the ultrathin thickness of F_{16}CuPc, hole tunnelling occurred in which F_{16}CuPc acted as a tunnelling channel, resulting in a large current (Figure S4d). It is worth

noting that over-barrier thermionic emission also contributes to the conduction process under small bias region.[4] In addition to the lack of the temperature dependence, this tunnelling-dominated transport behaviour can be further demonstrated and fitted by the Fowler–Nordheim model at 77 K, described as $I_{\mathrm{FNT}} \propto V^2 \exp(-\frac{8\pi d\sqrt{2m^*\varphi^3}}{3heV})$, which exhibits a linear relation with a negative slope of –27.9 (Figure S4b).[4, 5]

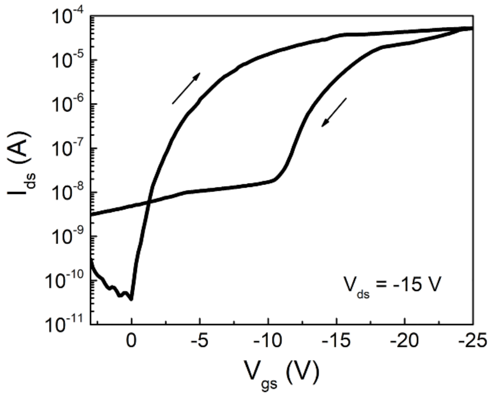

Figure S5. Transfer curve of the heterojunctions with double sweep at V_{ds} = –15V.

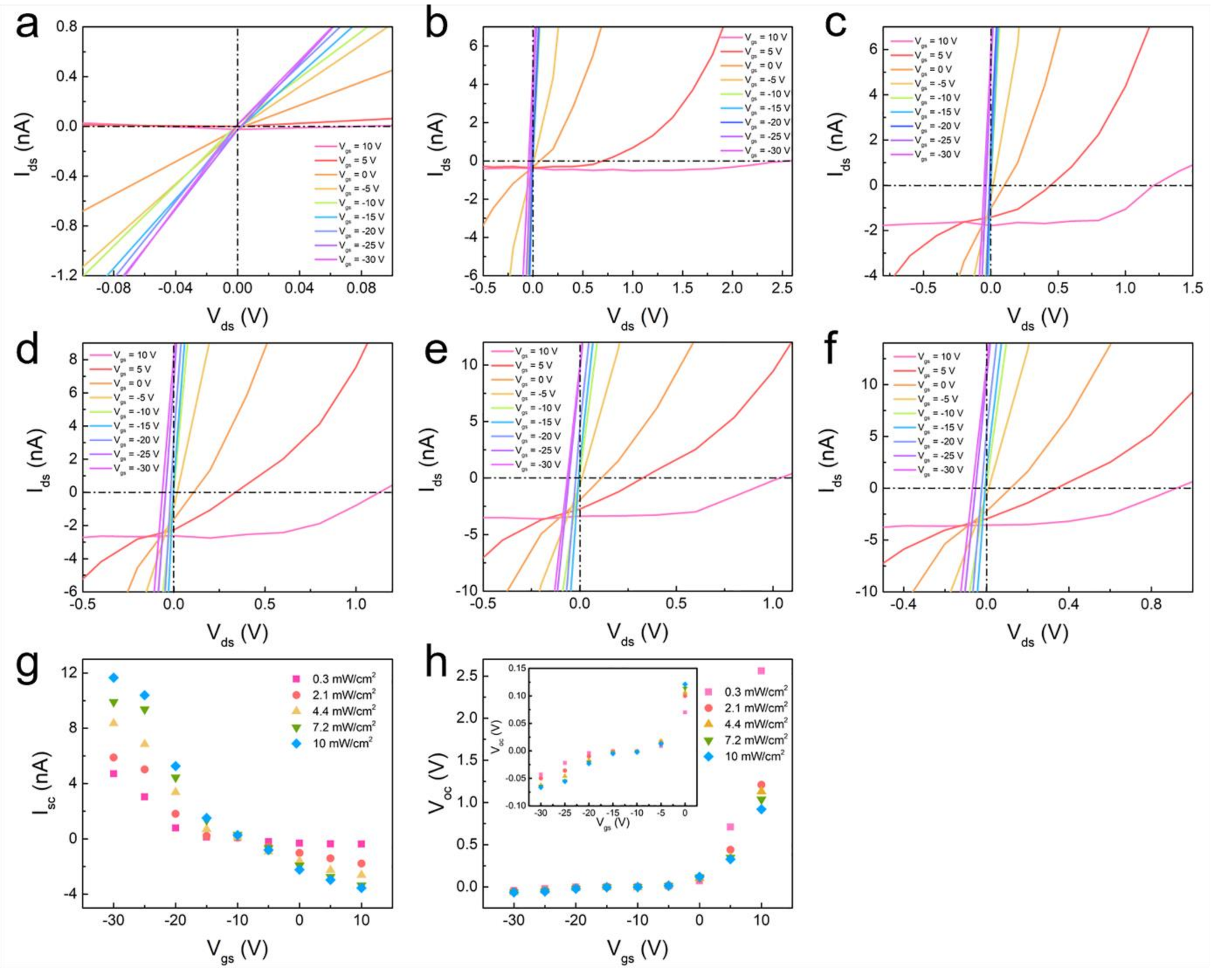

Figure S6. a) *I*−*V* curves in the dark under different gate voltages. b-f) *I*−*V* curves under various light intensities and different gate voltages. g) The short-circuit current versus the gate voltages under various light intensities. h) The open-circuit current versus the gate voltages under various light intensities.

Note S4. The gate-tunable photovoltaic properties of our asymmetric heterojunctions.

We measured gate-tunable photovoltaic properties (from 10 V to –30V) under dark and different UV light illumination ranging from 0.3 mW/cm^2 to 10 mW/cm^2. Subsequently, we extracted the gate-dependent short-circuit current (I_{sc}) and open-circuit voltage (V_{oc}) under different UV light illumination ranging from 0.3 mW/cm^2 to 10 mW/cm^2 (Figures S6g, h).

(1) In the dark, there is no obvious upward or downward movement of the output curves under different gate voltages from 10 V to –30V. Hence, no photovoltaic effect (I_{sc} and V_{oc}) can be observed (Figure S6a).

(2) Under different illumination power (Figures S6b-h):

When $V_{gs} \geq 0$ V, the curves are all downshifted, exhibiting a distinct photovoltaic response. When the devices were under light illumination, photoinduced electron-hole pairs were generated and separated in the heterojunction under the large built-in electric field, whose direction was the same as that of the interfacial dipoles. Note that, in our asymmetric heterojunction architecture, holes were collected on the F_{16}CuPc side, and electrons crossed the heterointerface and arrived at the C_8-BTBT side. Therefore, positive V_{oc} and negative I_{sc} were observed, which obviously improved with the increase of gate voltages.

When V_{gs} ranges from –5 V to –15 V, the gate voltage induced an electric field from F_{16}CuPc to C_8-BTBT, which was opposite to the direction of the interface dipole. In this region, we observed extremely small and indistinguishable V_{oc} and I_{sc}, which were both close to 0. In fact, there is a transition from negative current (positive voltage) to positive current (negative voltage) for I_{sc} (V_{oc}) when V_{gs} ranges from –5 V to –15 V, which means that the intensity of gate-induced electric field was approximately equivalent to that of the interface dipole (also, was a situation of flat band).

When $V_{gs} < -15$V, the curves are all upshifted. The direction of the gate-induced electric field was still different from that of the interfacial dipole, and the intensity of gate-induced electric field was larger than that of the interface dipole. Therefore, photoinduced electrons were collected on the F_{16}CuPc side, and photoinduced holes arrived at the C_8-BTBT side, which gave rise to negative V_{oc} and positive I_{sc}. Also, both V_{oc} and I_{sc} improved with the increase of gate voltage.

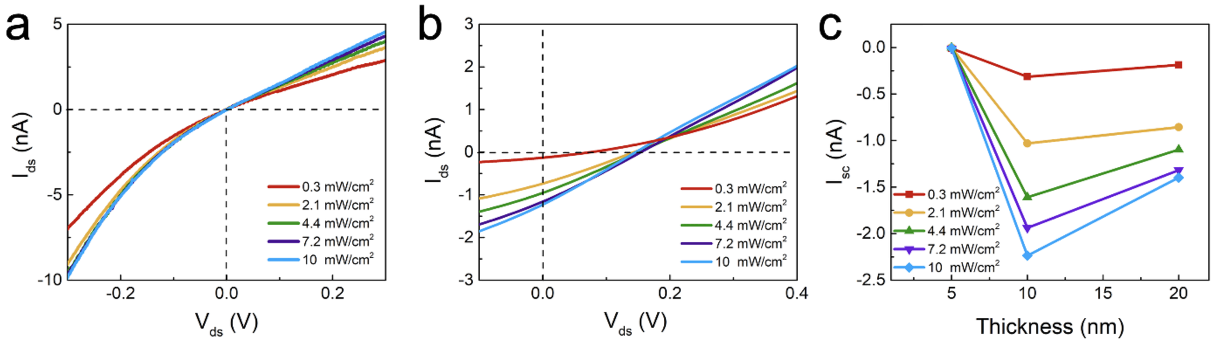

Figure S7. a) The photovoltaic response of our heterojunctions with 5 nm F_{16}CuPc. b) The photovoltaic response of our heterojunctions with 20 nm F_{16}CuPc. c) Relative short current response extracted from (a, b) and Figure 2b.

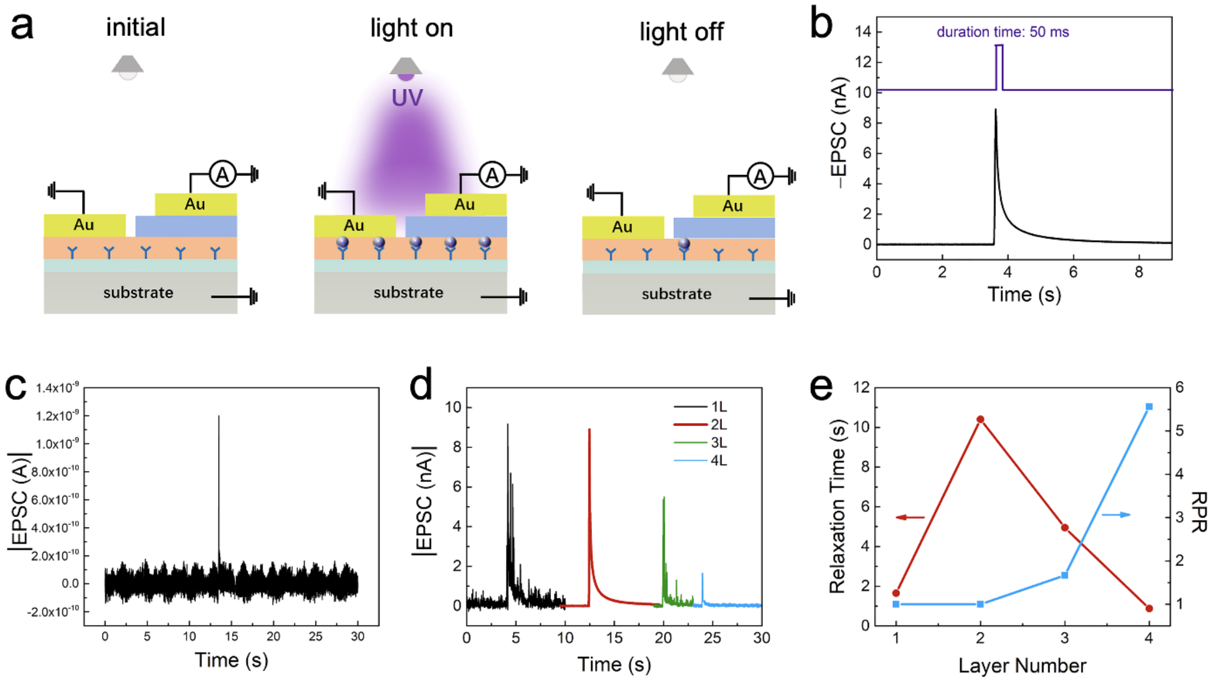

Figure S8. a) Schematic diagram of the self-powered synapse operation. b) The EPSC triggered by one light pulse with the duration time of 50 ms. c) The EPSC response of the C_8-BTBT/F_{16}CuPc heterojunction without the PMMA films. d) The C_8-BTBT layer-number-dependent EPSC response behaviours of the heterojunction under the same measurement conditions. e) Relative bilayer photocurrent response RPR and relaxation time extracted from d).

Note S5. Typical EPSC response and operation mechanisms of our self-powered optoelectronic synapses.

Initially, there was an extremely low current level in the heterojunction due to the lack of mobile charge carriers under dark conditions (the left panel of Figure S8a). When a light pulse was applied, the EPSC reached a peak value of ~9 nA because the charge trap states at the C_8-BTBT/PMMA interface were partly filled by the photoinduced electrons (Figure S8b, the middle panel of Figure S8a).[6–8] After switching off the light, the trapped electrons were gradually released and recombined with the photoinduced holes, giving rise to typical current decay and relaxation behaviours, which is in accordance with the short-term plasticity in

biological synapses (the right panel of Figure S8a). To further verify the interfacial charge trapping effect of PMMA, devices without PMMA were constructed. After the light pulse ends, the rapid decay of the EPSC implies that the interfacial charge trapping effect induced by PMMA has a vital impact on the slow decay of the current (Figure S8c). Additionally, we also demonstrated the C_8-BTBT-based thickness-dependent EPSC response. The bilayer C_8-BTBT devices demonstrated a slower decay rate and a larger current response, which is the optimized choice in our device (Figures S8d, e).

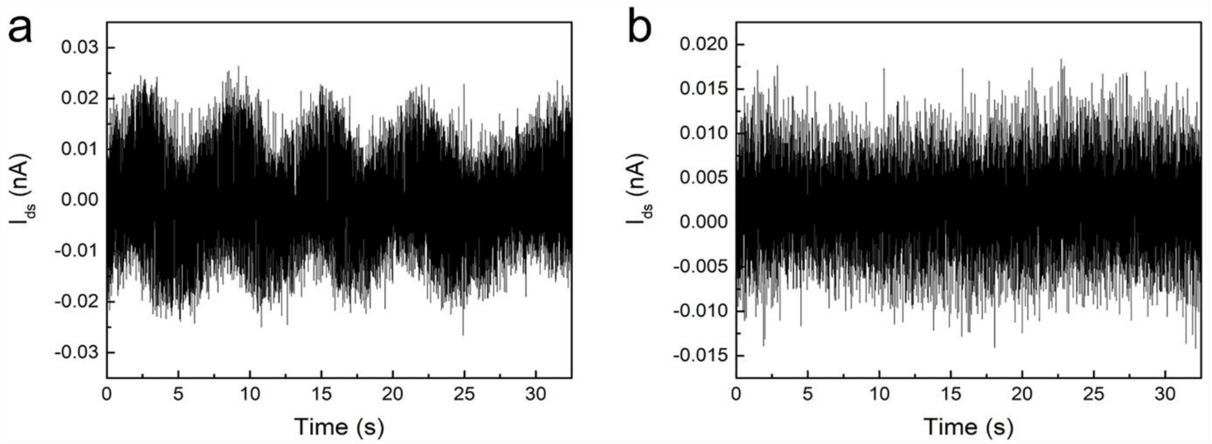

Figure S9. a) EPSC of the structure of PMMA/C_8-BTBT under various light illumination without any electrical bias. b) EPSC of the structure of PMMA/F_{16}CuPc under various light illumination without any electrical bias.

Note S6. EPSC response of the structure of PMMA/F_{16}CuPc and PMMA/C_8-BTBT.

We measured self-powered synaptic behaviours in the structure of PMMA/F_{16}CuPc and PMMA/C_8-BTBT, respectively (Figure S9). There is no EPSC response because they cannot provide any photovoltaic properties to act as an internal power source like the C_8-BTBT/F_{16}CuPc heterojunctions.

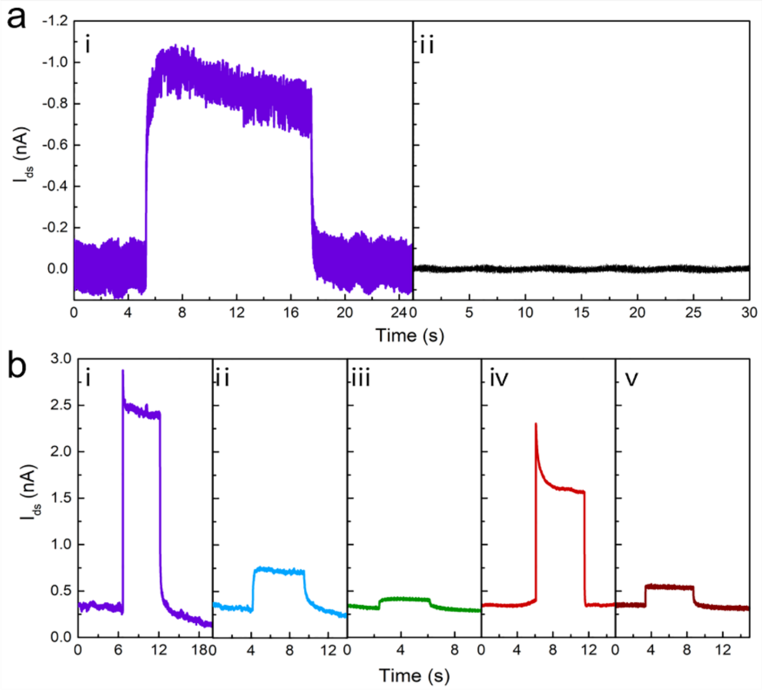

Figure S10. a) Transient photocurrent of the structure of PMMA/C_8-BTBT under various light illumination at V_{ds} = –0.1 V. b) Transient photocurrent of the structure of PMMA/F_{16}CuPc under various light illumination at V_{gs} = 20 V and V_{ds} = 10 V.

Note S7. The selective detection of UV light in the self-powered mode.

When the light absorption of the F_{16}CuPc/C_8-BTBT heterojunction is in the visible and infrared range, in which the photon energy is smaller than the band gap of C_8-BTBT but larger than that of F_{16}CuPc, photoinduced electron-hole pairs are only generated in F_{16}CuPc layers (Figure S1c and Figure S10). Note that in the self-powered mode, the holes transfer from F_{16}CuPc to C_8-BTBT, forming interfacial dipoles that partially impede the separation of the photoinduced electron-hole pairs under illumination.[9] Additionally, as the electron-rejection barrier (ΔLUMO = 2.9 eV) is larger than the hole-rejection barrier (ΔHOMO = 1.2 eV), electrons cross the heterointerface with a larger barrier blocking them than that for holes. Therefore, there is an ultralow photocurrent response in the self-powered mode.

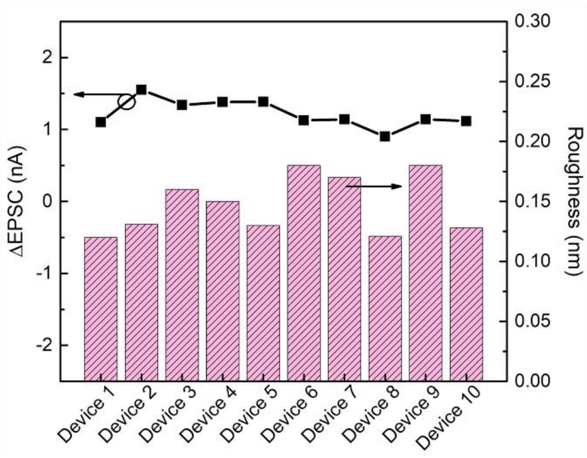

Figure S11. Repeatability of ΔEPSC and the roughness of C8-BTBT layers of 10 devices.

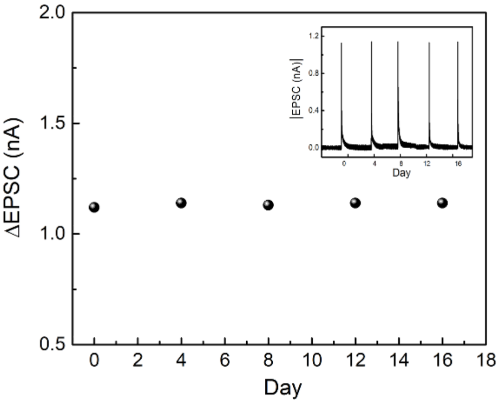

Figure S12. Stability of ΔEPSC in our self-powered optoelectronic synapses encapsulated by the Parylene films. The inset is the stability of EPSC in our self-powered optoelectronic synapses encapsulation by the Parylene films.

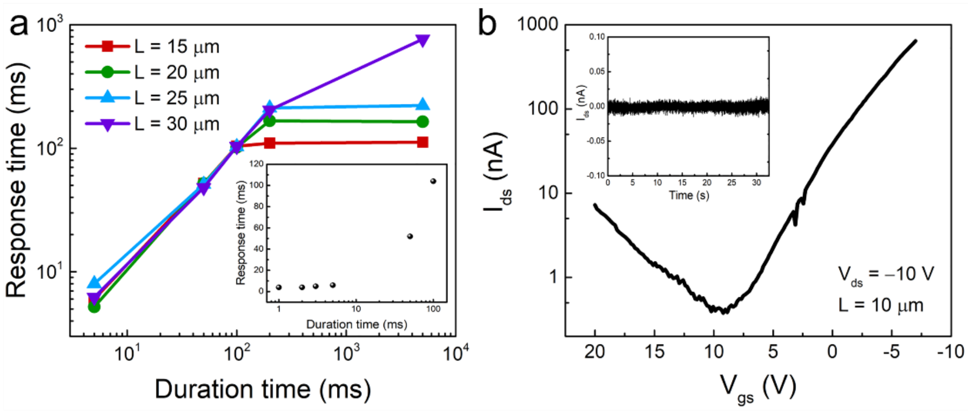

Figure S13. a) The response time versus the duration time of the UV pulse under different channel lengths in a self-powered mode. The inset is the response time versus the duration time of the UV pulse under L = 15 μm in a self-powered mode. b) The transfer behavior under L = 10 μm of our synaptic devices. The inset is the current response under L = 10 μm of our synaptic devices in a self-powered mode.

Note S8. The discussion of the limitation of the response time and the device footprint.

When applying a relatively short UV pulse (<100 ms), the response time of our devices under different channel length (L) is quite close to the duration time of the UV pulse. While further enlarging the duration time of the UV pulses, the current first reaches the saturated value within a short response time of ~110 ms in the synaptic devices under L = 15 μm. Subsequently, the response time for the current to reach saturation gradually increases (~160, 210, and 760 ms) with the increasing L (20, 25, and 30 μm) (Figure S13a). The channel length-dependent response time simultaneously indicated that the collection time of the photogenerated carriers increases with the increasing L. Furthermore, we measured the response time at L = 10 μm, however, there is no photocurrent response in a self-powered mode (inset of Figure S13b). We further examined the transfer properties of our devices (Figure S13b). An ambipolar transport behavior can be observed, which was caused by the blurring effect during the deposition of $F_{16}CuPc$.[10] In addition, we explored the ultimate

response time under the channel length of 15 μm and the shortest switching time of our devices was ~5 ms (inset of Figure S13a).

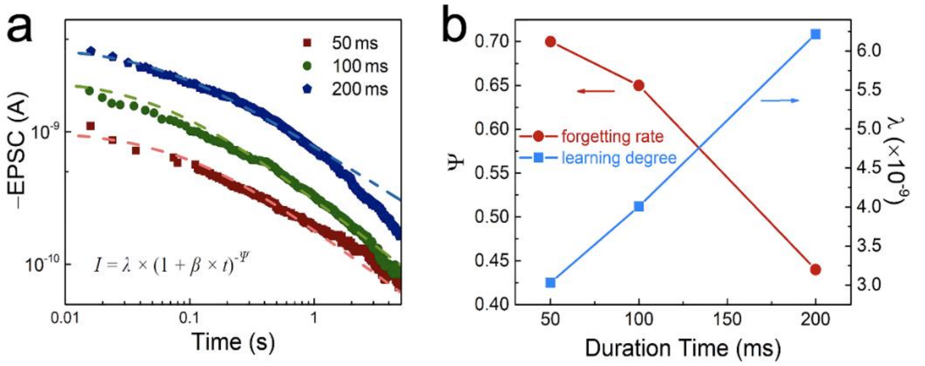

Figure S14. a) Current decay under various duration time is fitted by the Wickelgren's power law: $I = \lambda \times (1 + \beta \times t)^{-\Psi}$. The data are taken from the lower panel of Figure 3a. b) Changes in forgetting factor ψ as the duration time decreases and changes in learning degree λ as the duration time increases.

Note S9. The learning and forgetting characteristics of our self-powered optoelectronic synapses.

We defined the current increasing with the light pulses as "learning". After stopping irradiation, the process of current relaxation is defined as "forgetting". The forgetting (current decay) curves under light pulses (2.1 mW cm^{-2}, 365 nm) with different durations were fitted by Wickelgren's power law, which is described by the biological forgetting law:[11,12] $I = \lambda \times (1 + \beta \times t)^{-\Psi}$ (Figure S14a), where I is the memory level, λ is the initial degree of learning, β is a scaling parameter, and Ψ is the forgetting rate. We plotted λ and Ψ versus the duration of the light pulse in Figure S11b. With increasing duration of the light pulse, λ increases from 1.11×10^{-9} to 3.73×10^{-9}, and Ψ decreases from 0.8 to 0.5, indicating that repeated light pulses can increase the synaptic strength.

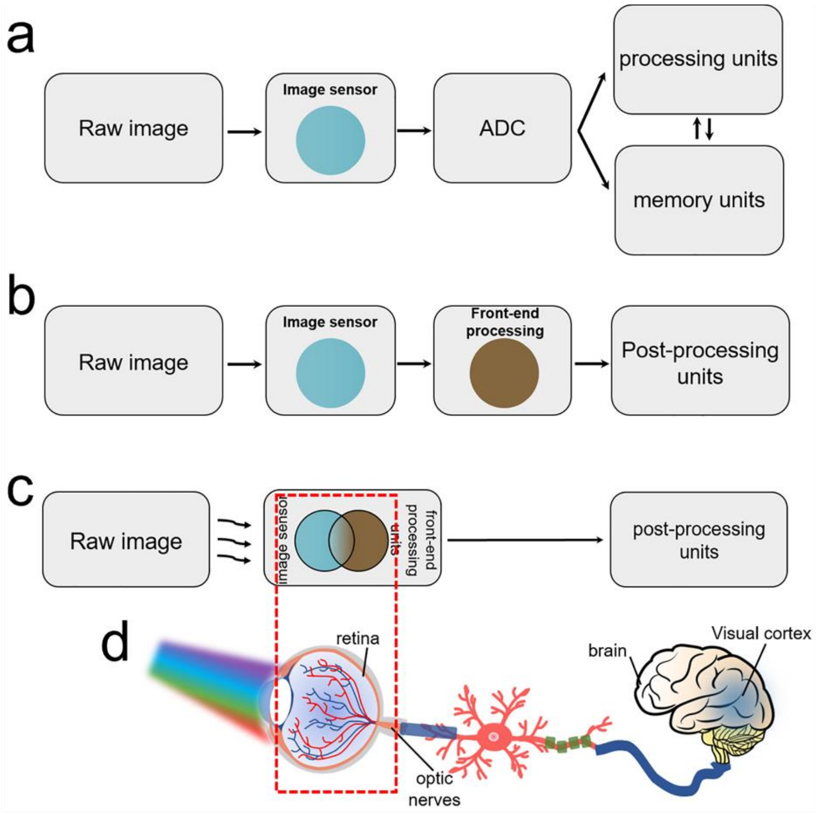

Figure S15. a) The structure of the conventional artificial visual system. b) The structure of the near-sensor computing artificial visual system. c) The structure of the in-sensor computing artificial visual system. d) Schematic of the human visual system.

Note S10. Comparison of different sensory computing architectures.

In the conventional CMOS-based sensory computing architecture, raw images are usually converted into analogue electrical signals through image sensors and then sent to processing units for digital computing through an analogue-to-digital converter (ADC) (Figure S15a).[13] In the near-sensor computing architecture, front-end processing units residing beside the image sensors can minimize the data transmission distance without the use of an ADC (Figure S15b).[13] In contrast, in the in-sensor computing architecture, image sensing and front-end processing units can be integrated in a single device for image preprocessing (Figure 3d and Figure S15c). Note that our simulated image preprocessing function (low-level processing)

usually occurs in the front-end processing units of an artificial visual system, also equivalent to the function of the retina of the human visual system (red box in Figures S15c, d).

Note S11. The importance of multispectral sensing and spectral selectivity capabilities in a single device.

Photodetectors, which are the vital elements for imaging and communication, are usually divided into broadband photodetectors and narrowband photodetectors. In general, broadband photodetectors without obvious color discrimination have been widely studied for various applications, such as imaging, communication, and sensing.[14] For example, Z. Zhao et al. designed double-layered organic photodetectors composed of one absorber layer and one multiplication layer to achieve a broad spectral response covering 350 to 950 nm without obvious color discrimination.[15] In contrast, narrowband photodetection with wavelength-selectivity is required for realizing color discrimination, which is potential for numerous fields.[16] While, these photodetectors usually operate exclusively in one regime—either in the broadband or in the narrowband—limiting their broader applications. Hence, realizing multifunctional photodetectors that can sense photons in both regions would introduce a new level of versatility to optoelectronics for broader applications.[16,17]

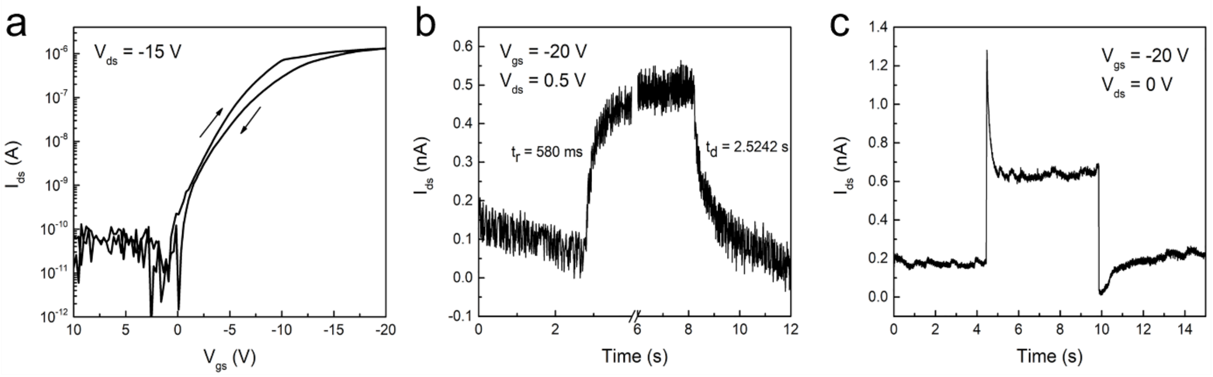

Figure S16. a) Transfer curves of the heterojunctions without PMMA layers with a double sweep at V_{ds} = –15V. b) Transient photocurrent of the heterojunctions without PMMA layers under UV light illumination at V_{gs} = –20 V and V_{ds} = 0.5 V. c) Transient photocurrent of the heterojunctions with PMMA layers under UV light illumination at V_{gs} = –20 V and V_{ds} = 0 V.

Note S12. The origin of the abrupt change in photoswitching time between UV and visible/near-infrared wavelengths.

To determine whether the origin of the abrupt change in photoswitching time between UV and visible/near-infrared wavelengths, we measured the transfer curves of our devices with/without PMMA layers through a double sweep. A significantly reduced hysteresis can be observed in the devices without PMMA layers, indicating that the electron trapping effect was weakened (Figure S16a). Subsequently, we measured the photocurrent response of devices without PMMA layers under UV light illumination at V_{ds} = 0.5 V and V_{gs} = −20 V (Figure S16b). The rise and decay times extracted from the photoswitching curves are 580 ms and 2.5 s, respectively. Although the photoswitching time is shorter than those in the devices with PMMA layers under UV light illumination, they are still longer than those in the devices with PMMA layers under visible/near-infrared light illumination, indicating that the PMMA layer is not the only decisive factor. Note that UV light has higher energy than visible/near-infrared light and can be simultaneously absorbed by both materials. In addition, we also measured the photocurrent response of devices with PMMA layers under UV light illumination at V_{ds} = 0 V

and V_{gs} = −20 V (Figure S16c). There was almost no relaxation behavior, indicating the role of the drain voltage (Note that the peak in Figure S16c may be the alternating current, arising from the alternating current photovoltaic effect[18]). Therefore, we considered that photogenerated carriers were trapped by deep-level traps for longer photoswitching time under the synergism of UV light and drain voltage.

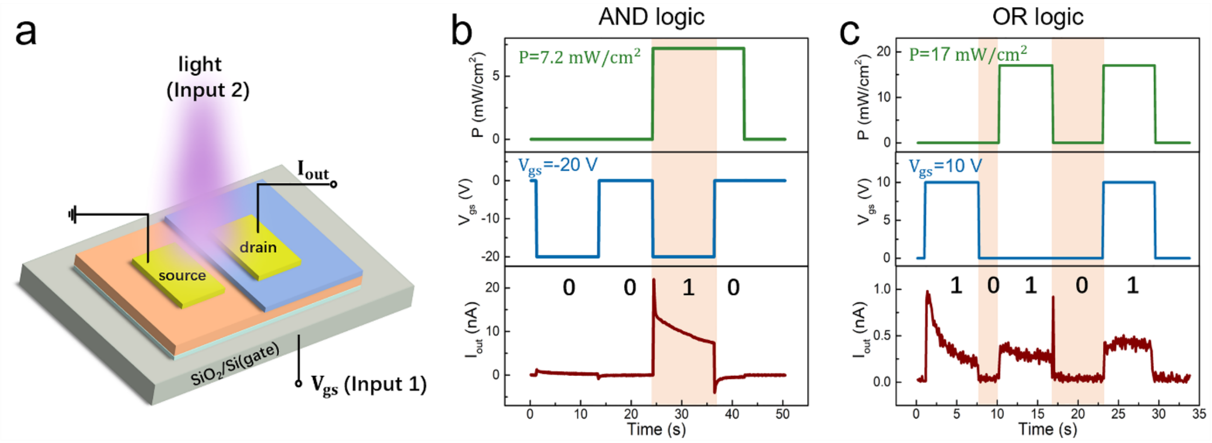

Figure S17. a) Schematic diagram and the measurement setup. b) Output of the AND logic for different input logic states versus time. c) Output of the OR logic for different input logic states versus time.

Note S13. Optoelectronic logic operations of our asymmetric heterojunctions.

The incident light and gate voltage are two different inputs (Input 1 and Input 2, respectively), and the current level (I_{out}) is the output signal (Figure S16a). Figure S16b shows the typical time trace of output signal I_{out} for AND logic operation. For the different input signals, we defined incident light on (7.2 mW cm^{-2}) and off as logic “1” and “0” and gate voltages of −20 V and 0 V as logic “1” and “0”. For the output signal results, we defined the high current level (~10 nA) and low current level (~80 pA) as logic “1” and “0”, which was proven to be an AND logic gate. To fulfil OR logic operation, suitable parameters of the input signals were selected. Furthermore, we confirmed the operation as a mixed optoelectronic OR gate, as shown in Figure S16c.

Table S1. The summary of the changes in the interfacial dipole and band alignment according to the gate and drain bias and the changes in the photo-induced and gate-induced photocurrent responses.

	V_{gs} > 0 V		V_{gs} = 0 V		V_{gs} < 0 V(<–15 V)	
	UV	blue to NIR	UV	blue to NIR	UV	blue to NIR
Band alignment	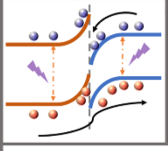	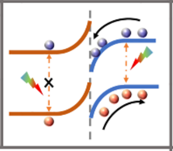	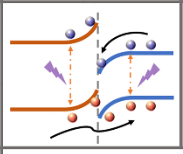	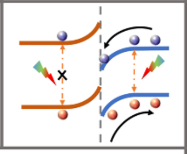	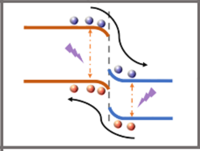	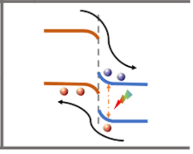
Device diagram	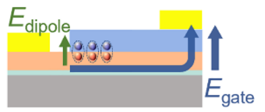		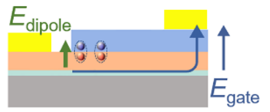		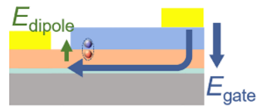	
V_{ds} = 0 V	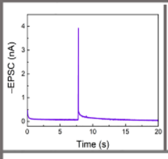	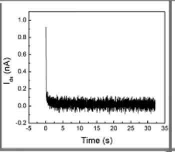	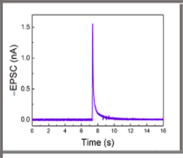	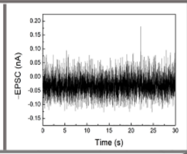	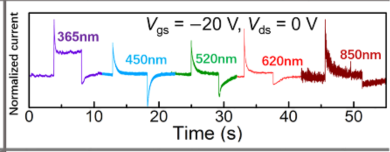	
V_{ds} = 0.5 V					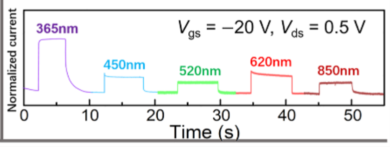	

When $V_{gs} \geq 0$ V and $V_{ds} = 0$ V, the direction of the gate-induced electric field was from the p-type semiconductor to the n-type semiconductor, which was the same as that of the initial interfacial dipole stemming from the hole transfer from $F_{16}CuPc$ to C_8-BTBT. Under UV light illumination, photoinduced electron-hole pairs were generated and separated in the heterojunction under the large built-in electric field (p-type to n-type). The short distance between C_8-BTBT and $F_{16}CuPc$ benefits the collection of the photogenerated holes, and the lateral channel of C_8-BTBT can contribute to the synaptic behaviours after the light pulse ends due to the photogenerated electron trapping at the C_8-BTBT/PMMA interface. A negative photocurrent response can be observed, which is consistent with the gate tunable photovoltaic response. While the light absorption of the $F_{16}CuPc/C_8$-BTBT heterojunction is in the visible and infrared range, in which the photon energy is smaller than the band gap of C_8-BTBT but larger than that of $F_{16}CuPc$, photoinduced electron-hole pairs are only generated

in $F_{16}CuPc$ layers. Note that interfacial dipoles could partially impede the separation of the photoinduced electron-hole pairs under illumination. Additionally, as the electron-rejection barrier (ΔLUMO = 2.9 eV) is larger than the hole-rejection barrier (ΔHOMO = 1.2 eV), electrons cross the heterointerface with a larger barrier blocking them than that for holes. Therefore, there is an ultralow photocurrent response.

When $V_{gs} < 0$ V and $V_{ds} = 0$ V, the direction of gate-induced electric field is different from that of the interfacial dipole, which can be beneficial to charge transfer at the type-II interface. By virtue of the absorption characteristics of the heterojunction, this gate tunability allows our devices with the ability of the multispectral sensing from UV to NIR regions. A positive photocurrent response can be observed, which is still consistent with the gate tunable photovoltaic response. Note that the photocurrent response is relatively low only with the gate voltage (i.e., the light response only comes from the photovoltaic effect), hence, we added a small drain voltage (V_{ds} = 0.5 V) in which the direction is the same as that of built-in electric field for more obvious photocurrent response.

References:

[1] P. Luo, F. Zhuge, F. Wang, L. Lian, K. Liu, J. Zhang, T. Zhai, *ACS Nano* **2019**, 13, 9028.

[2] X. Zhou, X. Hu, S. Zhou, H. Song, Q. Zhang, L. Pi, L. Li, H. Li, J. Lü, T. Zhai, *Adv. Mater.* **2018**, 30, 1703286.

[3] Y. Duan, J. Qian, J. Guo, S. Jiang, C. Yang, H. Wang, Q. Wang, Y. Shi, Y. Li, *Adv. Electron. Mater.* **2020**, 6, 2000438.

[4] R. Cheng, F. Wang, L. Yin, Z. Wang, Y. Wen, T. A. Shifa, J. He, *Nat. Electron.* **2018**, *1*, 356.

[5] X. Zhou, X. Hu, S. Zhou, H. Song, Q. Zhang, L. Pi, L. Li, H. Li, J. Lü, T. Zhai, *Adv. Mater.* **2018**, *30*, 1703286.

[6] W. Li, M. Yi, H. Ling, F. Guo, T. Wang, T. Yang, L. Xie, W. Huang, *J. Phys. D: Appl. Phys.* **2016**, *49*, 125104.

[7] L. Shang, Z. Ji, H. Wang, Y. Chen, X. Liu, M. Han, M. Liu, *IEEE Electron Device Lett.* **2011**, *32*, 1451.

[8] M. M. Rehman, B. S. Yang, Y. J. Yang, K. S. Karimov, K. H. Choi, *Curr. Appl. Phys.* **2017**, *17*, 533.

[9] P. Luo, F. Zhuge, F. Wang, L. Lian, K. Liu, J. Zhang, T. Zhai, *ACS Nano* **2019**, *13*, 9028.

[10] X. Ye, X. Zhao, S. Wang, Z. Wei, G. Lv, Y. Yang, Y. Tong, Q. Tang, Y. Liu, *ACS Nano* **2021**, *15*, 1155.

[11] J. T. Wixted, S. K. Carpenter, *Psychol. Sci.* **2007**, *18*, 133.

[12] X. B. Yin, R. Yang, K. H. Xue, Z. H. Tan, X. D. Zhang, X. S. Miao, X. Guo, *Phys. Chem. Chem. Phys.* **2016**, *18*, 31796.

[13] F. C. Zhou, Y. Chai, *Nat. Electron.* **2020**, *3*, 664.

[14] J. Miao, F. Zhang, *Laser Photonics Rev.* **2019**, *13*, 1800204.

[15] Z. Zhao, J. Wang, C. Xu, K. Yang, F. Zhao, K. Wang, X. Zhang, F. Zhang, J. *Phys. Chem. Lett.* **2020**, *11*, 366.

[16] M. I. Saidaminov, M. A. Haque, M. Savoie, A. L. Abdelhady, N. Cho, I. Dursun, U. Buttner, E. Alarousu, T. Wu, O. M. Bakr, *Adv. Mater.* **2016**, *28*, 8144.

[17] W. Wang, M. Du, M. Zhang, J. Miao, Y. Fang, F. Zhang, *Adv. Opt. Mater.* **2018**, *6*, 800249.

[18] H. Zou, G. Dai, A. C. Wang, X. Li, S. L. Zhang, W. Ding, L. Zhang, Y. Zhang, Z. L. Wang, *Adv. Mater.* **2020**, 32, 1907249.
